# Using the Patient Portal Sexual Health Instrument in Surveys and Patient Questionnaires Among Sexual Minority Men in the United States: Cross-sectional Psychometric Validation Study

**DOI:** 10.2196/18750

**Published:** 2021-02-10

**Authors:** Kevon-Mark P Jackman, Jeremy Kane, Hadi Kharrazi, Renee M Johnson, Carl Latkin

**Affiliations:** 1 Department of Mental Health Johns Hopkins Bloomberg School of Public Health Baltimore, MD United States; 2 Department of Epidemiology Columbia University Mailman School of Public Health NY, NY United States; 3 Department of Health Policy and Management Center for Population Health IT Johns Hopkins Bloomberg School of Public Health Batimore, MD United States; 4 Department of Health, Behavior, and Society Johns Hopkins Blooomberg School of Public Health Baltimore, MD United States

**Keywords:** health information technology, sexual behavior, HIV, STI, patient portals

## Abstract

**Background:**

Patient portal modules, including electronic personal health records, health education, and prescription refill ordering, may be leveraged to address the sexually transmitted infection (STI) burden, including HIV, among gay, bisexual, and other sexual minority men (SMM). Theoretical frameworks in the implementation sciences highlight examining constructs of innovation attributes and performance expectations as key determinants of behavioral intentions and the use of new web-based health technologies. However, behavioral intentions to use patient portals for HIV and other STI prevention and care among SMM is understudied.

**Objective:**

The aim of this study is to develop a brief instrument for measuring attitudes focused on using patient portals for STI prevention and care among a nationwide sample of SMM.

**Methods:**

A total of 12 items of the American Men’s Internet Survey-Patient Portal Sexual Health Instrument (AMIS-PPSHI) were adapted from a previous study. Psychometric analyses of the AMIS-PPSHI items were conducted among a randomized subset of 2018 AMIS participants reporting web-based access to their health records (N=1375). Parallel analysis and inspection of eigenvalues in a principal component analysis (PCA) informed factor retention in exploratory factor analysis (EFA). After EFA, Cronbach α was used to examine the internal consistency of the scale and its subscales. Confirmatory factor analysis (CFA) was used to assess the goodness of fit of the final factor structure. We calculated the total AMIS-PPSHI scale scores for comparisons within group categories, including age, STI diagnosis history, recency of testing, serious mental illness, and anticipated health care stigma.

**Results:**

The AMIS-PPSHI scale resulting from EFA consisted of 12 items and had good internal consistency (α=.84). The EFA suggested 3 subscales: sexual health engagement and awareness (α=.87), enhancing dyadic communication (α=.87), and managing sexual health care (α=.79). CFA demonstrated good fit in the 3-factor PPSHI structure: root mean square error of approximation=0.061, comparative fit index=0.964, Tucker-Lewis index=0.953, and standardized root mean square residual=0.041. The most notable differences were lower scores on the enhanced dyadic communication subscale among people living with HIV.

**Conclusions:**

PPSHI is a brief instrument with strong psychometric properties that may be adapted for use in large surveys and patient questionnaires in other settings. Scores demonstrate that patient portals are favorable web-based solutions to deliver health services focused on STI prevention and care among SMM in the United States. More attention is needed to address the privacy implications of interpersonal use of patient portals outside of traditional health settings among persons with HIV.

## Introduction

### Psychometrics in Health Technology Behavior Research

The use of digital strategies to address public health priorities, such as HIV, has increased tremendously in the United States in the last decade [1-4]. To inform implementation strategies, many survey instruments have been developed to measure contextual attitudes about health technology use across patient populations. The National Cancer Institute’s Health Information National Trends Survey (HINTS) is a biennial nationally representative survey to assess the impact of the health information environment. It includes items useful to describe individual perceptions about the privacy and security of electronic medical records in a national sample [5]. Instrument development has been applied to assess attitudes about health technology use in specific populations, such as among older adults [6]. Psychometric constructs assessed within instruments are sometimes the basis of path models developed to measure behavioral intentions among consumers using health information technology, such as the Health Information Technology Acceptance Model [7]. This study focuses on developing an instrument for a neglected area of study, patient portal use for the prevention and care of sexually transmitted infections (STIs), including HIV.

### Patient Portals

Patient portals provide patients with secure web-based access to health information, such as laboratory results and prescription medications. The web-based personal health information that a consumer accesses when using their patient portal is a type of health information technology (Health IT), referred to as personal health records (PHRs) [8,9]. Health IT is a broad concept that encompasses an array of technologies applied in health systems, such as PHRs, electronic health records (EHRs), or e-prescribing [10]. Logging on to a patient portal, users may message doctors, locate health education content, and a variety of options, depending on the Health IT platform. Certification for Health IT products is established by standards, implementation specifications, and certification criteria adopted by the US Secretary of Health and Human Services [11]. Data security and privacy measures, such as encrypting authentication credentials, are outlined within standards [12]. This is important because privacy concerns and mistrust are known barriers to PHR adoption [13,14]. Nevertheless, among US sexual minority men (SMM), patient portals are highly acceptable for delivery of comprehensive sexual health services that support HIV preventative behaviors, such as disclosing STI PHRs with sex partners [15,16].

In a 2016 market of approximately 186 certified vendors, 92% of US hospitals contracted either Cerner, MEDITECH, Epic Systems, CPSI, McKesson, and MEDHOST to supply certified Health IT [17]. To be certified, EHR vendors must include a patient portal module and use Fast Healthcare Interoperability Resources specifications to allow communication across vendor platforms [12,18]. Examples of patient portals are Epic’s MyChart, Veterans Administration’s My HealtheVet, Geisinger’s MyGeisinger, and Kareo’s Kareo EHR Patient Portal. There are over 300 unique Health IT products with 2015 certification that allow patients (ie, consumers) to view, transmit, and download patient medical data to a third party [19]. PHR systems may also be standalone and untethered to a health system’s EHR system [9].

As a result of federal incentive programs, namely, the Merit-Based Incentive Payment System programs Advancing Care Information, which have replaced Meaningful Use, patient portal access in health care settings has grown exponentially in the last decade and provides an excellent opportunity to deliver important health communications to patients [20-22]. By 2015, 95% of hospitals and 63% of office-based physicians had IT solutions allowing patients to view web-based health records [22,23]. Patient medical records are often perceived as highly private and only to be viewed by the patient and authorized representatives, which are based on important ethical and societal concerns of privacy [24-26]. However, portals also allow patients to interpersonally use information in PHRs outside of health care settings, such as sharing laboratory results or prescriptions with family, friends, and partners when promoting health behaviors [27]. Furthermore, few empirical studies have measured the preferences for and potential health benefits of the patient portal for sexual health and well-being on both individual and interpersonal levels.

### The Utility of Patient Portals for STIs

To date, little information is available on patients’ perceptions of patient portals and their potential use for the prevention and care of STIs, including HIV [28-30]. STIs are substantial public health problems, and they may be particularly well suited for prevention and management with patient portals. With billions of dollars in US health care costs, an estimated 20 million new infections each year, and as major causes of preventable disease, STIs are serious and growing public health threats [31]. In 2018, there were 2,457,118 combined cases of chlamydia, gonorrhea, and syphilis alone in the United States, continuing the trend of record-breaking numbers within the last decade [32]. The increasing prevalence of multidrug-resistant gonorrhea and the rise in syphilis infections also signal the urgent need for more effective prevention strategies [33,34].

Inefficient access and adherence to HIV medication among people living with HIV and pre-exposure prophylaxis (PrEP) among some SMM risk groups contribute to the burden of new HIV infections. In fact, persons with HIV who attain virologic suppression cannot transmit HIV to others, as regular PrEP use can effectively prevent the acquisition of HIV infection [35,36]. Adherence requires compliance with outpatient provider visits, filling in prescriptions, and taking antiretroviral therapy (ART) or PrEP over time; however, nonadherence can increase HIV disease progression and risk of disease transmission. Patient portals may effectively be able to create an easy-to-use system for organizing health information and be helpful in engaging SMM in care on all fronts of HIV infection.

Given the stigma about same-sex sexuality and talking about HIV and STIs, portals can also bridge communication gaps with providers and sex partners. According to the Centers for Disease Control and Prevention, gay, bisexual, and other SMM are disproportionately impacted by syphilis, HIV, and other STIs [37,38]. Factors such as patient-provider communication barriers and anticipated health care stigma experienced among SMM are known to hinder the use of STI preventive services such as testing [39-41]. In addition, controlling HIV is harder among persons with mental health issues, resulting in lower rates of HIV virologic suppression [42,43]. In a call to action for comprehensive HIV services for SMM, Beyrer et al [44] recommend health providers to provide integrated mental health services for SMM. SMM today have demonstrated high acceptability of medical and public health practice supported by mobile devices or mobile health (mHealth) in HIV interventions; however, these solutions have largely operated outside of the Health IT aegis of EHR and integrated PHR systems [45]. Previous studies examining perceptions of patient portal use for HIV care and STI prevention have demonstrated high acceptability and concern about potential breaches of privacy [28,30,46].

### Study Purpose

With this changing environment, new studies continue to burgeon modeling the implementation of health innovations for behavioral interventions focused on SMM. However, little data are available on the attributes and expectations of patient portals among SMM broadly across the United States. Theoretical frameworks, such as the Diffusion of Innovation theory and the extended unified theory of adoption and use of technology, are often used in behavioral sciences to inform implementation of new health technologies across consumer populations [6,47,48]. The theories posit that attributes of health technology and performance expectations are drivers of behavioral intentions to use health technology, and further behavioral intentions and habits determine behaviors or patterns of use. At the foundation of optimizing the design and uptake of new health technologies is scaling consumer perceptions about technology. Therefore, the overall goal of this study is to evaluate the psychometrics and adaptation of an instrument for measuring attitudes about using patient portals for STI prevention and care. Within-group comparisons of instrument scale scores are examined for categorical variables, including age, US region, last STI test, STI diagnosis, anticipated health care stigma, and mental health status.

## Methods

### Study Overview

Data were obtained from the 2018 American Men’s Internet Survey (AMIS). AMIS is an annual cross-sectional web-based survey of US residents who are aged at least 15 years; are cisgender male; and are gay or bisexual or have ever had sex with a man [49]. The study was conducted in compliance with federal regulations governing the protection of human subjects, and the study protocol was reviewed and approved by the Institutional Review Board at Emory University.

A randomized subset of 4647 AMIS participants was presented with survey items focusing on using patient portal services for STI prevention and care. Only survey participants reporting the ability to view their web-based health records were included in the analysis. Of these 2566 participants with patient portal access, the participants with *do not know* and *refuse to answer* responses on patient portal items were excluded from the analysis, resulting in a final analytic sample of 1375 men.

### Measures and Instrument Development

The Patient Portal Sexual Health Instrument (PPSHI) is a scale designed to evaluate perceptions of using patient portals to promote STI prevention and care behaviors. Items were originally adapted from a study among coed students at a historically Black university characterizing the perceived role of patient portals in supporting STI prevention behaviors—the Electronic Sexual Health Information Notification and Education (eSHINE) Study. In total, the eSHINE Study-Patient Portal Instrument (eSHINE-PPI) consisted of 19 items representing 4 subscales: (1) sexual health engagement (4 items), (2) informational resource compatibility (3 items), (3) valuation of services (5 items), and (4) PHR impact (7 items). A complete list of eSHINE-PPI items, factor loadings corresponding to unique subscales, and reliability coefficients can be found in Multimedia Appendix 1 [29,50]. To explore a more parsimonious instrument, we adapted 12 items from 3 eSHINE-PPI subscales to a nationwide survey of SMM. We focused on eliminating the eSHINE-PPI subscale items with the lowest factor loadings. Responses were also reduced from 7-point Likert scales to 4-point ordinal scales. We then adapted the eSHINE-PPI instrument to the AMIS-2018 survey.

### Adapting the Sexual Health Engagement Subscale

The lowest loading item on the sexual health engagement subscale, “I plan to manage my medical records with PHRs in the future,” was eliminated. The final adapted subscale (3 items) measured perceived attributes of using (vs not using) patient portals, specifically that (1) it is a more convenient way to manage my sexual health records, (2) it encourages people to be more aware of their sexual health, and (3) it will help people like me make better sexual health decisions. Responses were coded as follows: 0=strongly disagree, 1=disagree, 2=agree, and 3=strongly agree. Possible scores ranged from 0 to 9.

### Adapting the PHR Impact Subscale

The following 4 items were eliminated from the PHR impact subscale: (1) *PHRs make it easier for people to routinely have check-in conversations with partners about STI prevention*, (2) *Partners using PHRs will start talking about STI prevention earlier in a relationship*, (3) *I would have more discussions with partners about STI testing if PHRs were more commonly used*, and (4) *Using PHRs with a partner builds trust*. The final adapted subscale (3 items) measured agreement with beliefs that sharing STI PHRs with partners will (1) improve communication on HIV and other STIs, (2) improve confidence in the testing information a partner share, and (3) improve control over my sexual health and decision making. These responses were coded as follows: 0=definitely not, 1=probably not, 2=probably, and 3=definitely. Possible scores ranged from 0 to 9.

### Adapting the Valuation of Services Subscale

The item *In addition to electronic sexually transmitted disease (STD) results, which services are important for PHRs to include: Access to all of your medical records* was eliminated from the valuation of services subscale. Given the increase in telehealth success, the *Services to communicate with your doctor or health professionals* item was modified to specify *video chat services to communicate with health care providers* [51,52]. In response to recent decade increases in the use of interactive games to improve HIV prevention behaviors and the use of home test kits for STI screening, we added 2 items assessing valuation for *games to promote sexual health* and the *ability to order home test kits for HIV and STDs* to the valuation of services subscale [45,53-58]. For the final adapted subscale (6 items), participants were asked to rate the value of 6 patient portal features: (1) games to promote sexual health, (2) ability to order home test kits for STIs, (3) counseling and resources for people with STIs, (4) telehealth services, (5) ability to locate STI testing centers, and (6) tips or tools for managing sexual health. Responses on patient portal functionality items were coded as follows: 0=no value, 1=low value, 2=moderate value, and 4=high value. Possible scores ranged from 0 to 18.

The Informational Resource Compatibility Subscale (3 items) was not adapted to AMIS. Possible scores for the total AMIS-PPSHI ranged from 0 to 36.

### Statistical Analysis

First, we conducted a principal component analysis (PCA) of the 12 items to estimate an appropriate number of factors to retain. The retention of factors was determined by a parallel analysis and examining eigenvalues greater than 1.0 [59]. We used the exploratory factor analysis (EFA) to estimate the factor structure and loadings. Item loadings were examined for each factor using cutoff values ≥0.50. Sampling adequacy was indicated by a Kaiser-Meyer-Olkin (KMO) score greater than 0.80 [60].

We estimated the overall scale and subscale internal reliability with Cronbach α for the new AMIS-PPSHI. Confirmatory factor analysis (CFA) was used to evaluate measures of fit, including the Tucker-Lewis index (TLI), the comparative fit index (CFI), the root mean square error of approximation (RMSEA), and the standardized root mean square residual (SRMR). Thresholds for fit statistics were values close to 0.95 for TLI and CFI, values close to 0.08 for SRMR, and values close to 0.06 for RMSEA [61]. For standardized factor loadings, we used a cutoff of 0.50 [62].

We calculated the total AMIS-PPSHI scale scores for comparisons within group categories, including age, STI diagnosis history, recency of testing, serious mental illness, and anticipated health care stigma. The Kessler 6-item (K6) scale for psychological distress was used to determine the presence of serious mental illness (score 13 or greater) [63]. Anticipated health care stigma was defined as whether the participant was ever afraid to seek health care services because of worrying that someone may learn they have sex with men. Two-sample *t* tests were used to compare binary group differences in scale scores using a *P* value of .05 as the criterion for statistical significance. Analysis of variance (ANOVA) was used to compare group differences in scale scores for variables with more than 2 groups, also using a *P* value of .05 as the criterion for statistical significance.

## Results

### Sample Description

The sample consisted of 1375 US SMM with a median age of 34 years and an IQR of 25 to 50 years. The demographic characteristics of the study sample are presented in Table 1. Table 2 presents the items included in the questionnaire with calculated mean and mode scores. The KMO score (0.8492) established sampling adequacy. We used a Promax rotation because the factor correlations exceeded 0.32 [64]. On the basis of eigenvalues above 1.0 and parallel analysis, we estimated a 3-factor solution for factor analysis. Factor analysis yielded 3 distinct factors using the 0.50 cutoff and no cross-loading above 0.15.

**Table 1:** Demographic data for the study population, including age, region, and sexually transmitted infection diagnosis history, American Men’s Internet Survey 2018 (N=1375).

Category		Participants, n (%)	
**Age (years)**			
	15-24		318 (23.13)
	25-29		214 (15.56)
	30-39		291 (21.16)
	≥40		552 (40.15)
**US region**			
	Northeast (eg, New York and Vermont)		221 (16.07)
	Midwest (eg, Illinois and Ohio)		314 (22.84)
	South (eg, Florida and Alabama)		505 (36.73)
	West (eg, California and Oregon)		335 (24.36)
**Race or ethnicity**			
	Black, non-Hispanic		75 (5.51)
	Hispanic		181 (13.30)
	White, non-Hispanic		1020 (74.94)
	Other or multiple race		85 (6.25)
**History of STI^a^** **diagnosis**			
	No history of HIV or other STI diagnosis		1087 (79.05)
	Living with HIV		118 (8.58)
	Recent gonorrhea, chlamydia, or syphilis (and not living with HIV)		170 (12.36)
**STI test in the 12 months before the study**			
	No		1128 (82.04)
	Yes		247 (17.96)
**Willing to access web-based STI test results**			
	No		14 (1.02)
	Yes		1361 (98.98)

^a^STI: sexually transmitted infection.

**Table 2:** Descriptive statistics of suggested instrument items, American Men’s Internet Survey 2018 (N=1375).

Questionnaire item		Mean (SD)	Mode
**Patient engagement with sexual health care items**			
	Item 1. It is a more convenient way to manage my sexual health records.	2.56 (0.59)	3
	Item 2. It encourages people to be more aware of their sexual health.	2.52 (0.60)	3
	Item 3. It will help people like me make better sexual health decisions.	2.37 (0.71)	2
**Dyadic communication items**			
	Item 4. Improve communication on HIV and other sexually transmitted infections	2.49 (0.65)	3
	Item 5. Improve my confidence in the testing information a partner shares with me	2.54 (0.63)	3
	Item 6. Improve control over my sexual health and decision making	2.54 (0.64)	3
**Conceptual features (functionality)**			
	Item 7. Tips or tools for managing sexual health	2.43 (0.68)	3
	Item 8. Ability to locate STD^a^ test centers and services	2.63 (0.64)	3
	Item 9. Video chat for communicating with health care providers	2.16 (0.85)	2
	Item 10. Counseling and resources for people with STDs	2.54 (0.68)	3
	Item 11. Ability to order home test kits for HIV and STDs	2.59 (0.68)	3
	Item 12. Games to promote sexual health	1.54 (1.02)	1

^a^STD: sexually transmitted disease.

### The AMIS-PPSHI Structural Model

Table 3 displays the 12 items and their respective loadings across the 3 factors. Items 1-3 loaded on a factor named *sexual health engagement and awareness*, reflecting the perceived attributes of using patient portals to engage in sexual health care. Items 4-6 loaded on a factor named *enhancing dyadic communication*, reflecting the perceived attributes of using STI PHRs to share testing history with partners. Items 7-12 loaded on a third factor named *managing sexual health care*, reflecting the desired functionality of patient portals. Inter-factor correlation were 0.42 between the *sexual health engagement and awareness* and *enhancing dyadic communication* factors. *Sexual health engagement and awareness* and *managing sexual health care* had a correlation of 0.29. The estimated correlation between *managing sexual health care* and *enhancing dyadic communication* was 0.42. The CFA results (Table 4) suggested good fit indices for the 3-factor model (RMSEA=0.061, CFI=0.964, TLI=0.953, SRMR=0.041, and coefficient of determination =0.996).

**Table 3:** Exploratory factor analysis loadings and Cronbach α values, American Men’s Internet Survey 2018 (N=1375).

Questionnaire item	Factor loadings^a,b,c^		
	Factor 1. Sexual health engagement and awareness	Factor 2. Enhancing dyadic communication	Factor 3. Managing sexual health care
Item 1. It is a more convenient way to manage my sexual health records	*0.7994*	−0.0268	−0.0061
Item 2. It encourages people to be more aware of their sexual health	*0.8740*	−0.0231	0.0123
Item 3. It will help people like me make better sexual health decisions	*0.7374*	0.1093	0.0224
Item 4. Improve communication on HIV and other sexually transmitted infections	−0.0171	*0.* *7773*	0.0465
Item 5. Improve my confidence in the testing information a partner shares with me	0.0012	*0.8213*	−0.0123
Item 6. Improve control over my sexual health and decision making	0.0494	*0.8047*	−0.0226
Item 7. Tips or tools for managing sexual health	0.0806	0.1323	*0.5531*
Item 8. Ability to locate STD^d^ test centers and services	−0.0058	−0.0348	*0.6967*
Item 9. Video chat for communicating with health care providers	0.0199	−0.0478	*0.6382*
Item 10. Counseling and resources for people with STDs	0.0208	0.0039	*0.7415*
Item 11. Ability to order home test kits for HIV and STDs	−0.0202	−0.0057	*0.5621*
Item 12. Games to promote sexual health	−0.0743	0.0741	*0.5269*

^a^Factor loadings above 0.5000 are italicized.

^b^Cronbach α for factors: factor 1, sexual health engagement and awareness, α=.8678; factor 2, enhancing dyadic communication, α=.8689; and Factor 3, managing sexual health care, α=.7888.

^c^Interfactor correlations: r_factor1,factor2_=0.42, r_factor1,factor3_=0.29, and r_factor2,factor3_=0.42.

^d^STD: sexually transmitted disease.

**Table 4:** Confirmatory factor analysis of standardized factor loadings for Patient Portal Sexual Health Instrument, American Men’s Internet Survey 2018 (N=1375).

Questionnaire item	Factor loadings		
	Sexual health engagement and awareness	Enhancing dyadic communication	Managing sexual health care
Item 1. It is a more convenient way to manage my sexual health records	0.7929	N/A^a^	N/A
Item 2. It encourages people to be more aware of their sexual health	N/A	N/A	N/A
Item 3. It will help people like me make better sexual health decisions	N/A	N/A	N/A
Item 4. Improve communication on HIV and other sexually transmitted infections	N/A	0.8119	N/A
Item 5. Improve my confidence in the testing information a partner shares with me	N/A	0.8429	N/A
Item 6. Improve control over my sexual health and decision making	N/A	0.8357	N/A
Item 7. Tips or tools for managing sexual health	N/A	N/A	0.6633
Item 8. Ability to locate STD^b^ test centers and services	N/A	N/A	0.6840
Item 9. Video chat for communicating with health care providers	N/A	N/A	0.6202
Item 10. Counseling and resources for people with STDs	N/A	N/A	0.7760
Item 11. Ability to order home test kits for HIV and STDs	N/A	N/A	0.5493
Item 12. Games to promote sexual health	N/A	N/A	0.5339

^a^N/A: not applicable.

^b^STD: sexually transmitted disease.

### AMIS-PPSHI Scores

Table 5 presents factor scores for AMIS-PPSHI total and Table 6 factor scores for AMIS-PPSHI subscales by group categories using the sum of scores for variables with factor loadings above a cutoff of 0.50 [65]. The mean (M) and standard deviation (SD) AMIS-PPSHI total score was mean 28.94 (SD 5.14). Mean PPSHI subscale scores were as follows: sexual health engagement and awareness mean 7.46 (SD 1.70), enhancing dyadic communication mean 7.57 (SD 1.71), and managing sexual health care mean 13.90 (SD 3.22). By region, AMIS-PPSHI total scores were moderately higher than average in the South and West compared with the Northeast and Midwest regions. Mean AMIS-PPSHI scores decreased with increasing age category, most notably in the *enhancing dyadic communication* scale (F_3,1371_=10.87; *P*<.001). Participants who were tested 12 months before the study had slightly higher mean scores on sexual health engagement and awareness, mean 7.52 (SD 1.66) versus mean 7.18 (SD 1.83), and enhancing dyadic communication, mean 7.62 (SD 1.69) versus mean 7.38 (SD 1.82). The largest difference in the AMIS-PPSHI score were in comparisons of participants according to their history of HIV or recent STI. The overall highest scores were among people without HIV and without recent STI. Participants living with HIV have the lowest overall AMIS-PPSHI mean scores, mean 27.57 (SD 5.89), primarily because of enhancing dyadic communication scores, mean 6.85 (SD 2.13). There are no significant differences in scores by anticipated health care stigma nor serious mental illness. However, scores were marginally higher among participants with a Kessler 6-item psychological distress scale (K6) score≥13.

**Table 5:** Psychometrics of the Patient Portal Sexual Health Instrument by group, American Men’s Internet Survey 2018 (N=1375).

Group		AMIS-PPSHI^a^						
		Total						
		Cronbach α	Mean (SD)	Test statistic^b^			*P* value	
				*F* test (df)	*t* test (df)			
All (N=1375)		.8430	28.94 (5.14)	N/A^c^	N/A		N/A	
**US region**				4.37 (3,1371)	N/A		.005	
	Northeast (eg, New York and Vermont; n=221)	.8208	28.35 (4.88)					
	Midwest (eg, Illinois and Ohio; n=314)	.8529	28.29 (5.46)					
	South (eg, Florida and Alabama; n=505)	.8461	29.39 (5.13)					
	West (eg, California and Oregon; n=335)	.8378	29.24 (4.92)					
**Age category**				6.11 (3,1371)	N/A		<.001	
	15-24 (n=318)	.8149	29.52 (4.62)					
	25-29 (n=214)	.8095	29.44 (4.57)					
	30-39 (n=291)	.8422	29.28 (5.03)					
	≥40 (n=552)	.8608	28.22 (5.60)					
**STI^d^ test in 12 months before study**				N/A	1.64 (1373)		.10	
	Yes (n=1,128)	.8403	29.04 (5.10)					
	No (n=247)	.8548	28.45 (5.28)					
**History of STI diagnosis**				7.89 (2,1372)	N/A		<.001	
	No history of STI diagnosis (n=1087)	.8385	29.21 (4.98)					
	Living with HIV (n=118)	.8617	27.57 (5.89)					
	Recent gonorrhea, chlamydia, or syphilis and no HIV (n=170)	.8437	28.13 (5.36)					
**Anticipated health care stigma**				N/A	0.22 (1373)		.83	
	No (n=1037)	.8431	28.95 (5.18)					
	Yes (n=338)	.8433	28.88 (5.02)					
**Serious mental illness**				N/A	1.76 (1373)		.08	
	No (K6^e^<13; n=1113)	.8455	28.82 (5.20)					
	Yes (K6≥13; n=262)	.8288	29.44 (4.82)					

^a^American Men’s Internet Survey-Patient Portal Sexual Health Instrument.

^b^Test statistic: for analysis of variance (ANOVA) the F-value _(degrees of freedom groups, degrees of freedom residuals)_ test statistic is reported; for *t* tests, the *t* value (degrees of freedom) test statistic is reported.

^c^N/A: not applicable.

^d^STI: sexually transmitted infection.

^e^K6 refers to the Kessler 6-item psychological distress scale.

**Table 6:** Psychometrics of the Patient Portal Sexual Health Instrument Subscales, by group, American Men’s Internet Survey 2018 (N=1375).

Group		AMIS-PPSHI^a^ Subscales											
		Sexual health engagement and awareness				Enhancing dyadic communication				Managing sexual health care			
		Mean (SD)	Test statistic^b^		*P* value	Mean (SD)	Test statistics^b^		*P* value	Mean (SD)	Test statistic^b^		*P* value
			*F* test (df)	*t* test (df)			*F* test (df)	*t* test (df)			*F* test (df)	*t* test (df)	
		7.46 (1.70)	N/A	N/A	N/A	7.57 (1.71)	N/A	N/A	N/A	13.90 (3.22)	N/A	N/A	N/A
**US region**			2.41 (3,1371)	N/A	.07		0.75 (3,1371)	N/A	.52		5.63 (3,1371)	N/A	<.001
	Northeast (eg, New York and Vermont; n=221)	7.36 (1.75)				7.43 (1.68)				13.56 (3.02)			
	Midwest (eg, Illinois and Ohio; n=314)	7.30 (1.79)				7.55 (1.77)				13.43 (3.40)			
	South (eg, Florida and Alabama; n=505)	7.48 (1.70)				7.63 (1.67)				14.29 (3.25)			
	West (eg, California and Oregon; n=335)	7.64 (1.55)				7.60 (1.73)				14.00 (3.06)			
**Age category**			4.64 (3,1371)	N/A	.003		10.87 (3,1371)	N/A	<.001		1.47 (3,1371)	N/A	.22
	15-24 (n=318)	7.51 (1.55)				7.86 (1.38)				14.15 (3.20)			
	25-29 (n=214)	7.70 (1.44)				7.78 (1.52)				13.96 (2.99)			
	30-39 (n=291)	7.59 (1.65)				7.70 (1.64)				13.99 (3.09)			
	≥40 (n=552)	7.26 (1.87)				7.26 (1.93)				13.70 (3.38)			
**STI^d^ test in 12 months before study**			N/A	2.86 (1373)	.004		N/A	1.99 (1373)	.05		N/A	0.09 (1373)	.93
	Yes (n=1,128)	7.52 (1.66)				7.62 (1.69)				13.91 (3.23)			
	No (n=247)	7.18 (1.83)				7.38 (1.82)				13.89 (3.18)			
**History of STI diagnosis**			1.02 (2,1372)	N/A	.36		12.25 (2,1372)	N/A	<.001		6.13 (2,1372)	N/A	.002
	No history of STI diagnosis (n=1087)	7.49 (1.68)				7.66 (1.64)				14.06 (3.13)			
	Living with HIV (n=118)	7.39 (1.72)				6.85 (2.13)				13.33 (3.64)			
	Recent gonorrhea, chlamydia, or syphilis and no HIV (n=170)	7.30 (1.78)				7.52 (1.74)				13.31 (3.41)			
**Anticipated health care stigma**			N/A	0.47 (1373)	.64		N/A	1.02 (1373)	.31		N/A	0.35 (1373)	.73
	No (n=1037)	7.47 (1.70)				7.60 (1.74)				13.89 (3.26)			
	Yes (n=338)	7.42 (1.67)				7.49 (1.63)				13.96 (3.11)			
**Serious mental illness**			N/A	0.68 (1373)	.49		N/A	1.79 (1373)	.07		N/A	1.58 (1373)	.11
	No (K6^e^<13; n=1113)	7.44 (1.72)				7.53 (1.75)				13.84 (3.23)			
	Yes (K6≥13; n=262)	7.52 (1.61)				7.74 (1.51)				14.19 (3.17)			

^a^American Men’s Internet Survey-Patient Portal Sexual Health Instrument.

^b^Test statistic: for analysis of variance (ANOVA) the F-value _(degrees of freedom groups, degrees of freedom residuals)_ test statistic is reported; for *t* tests, the *t* value (degrees of freedom) test statistic is reported.

^c^N/A: not applicable.

^d^STI: sexually transmitted infection.

^e^K6 refers to the Kessler 6-item psychological distress scale.

## Discussion

### Principal Findings

The goal of this study is to develop a brief instrument for measuring attitudes focused on using patient portals for STI prevention and care among a nationwide sample of SMM. HIV and other STIs are costly and have a high burden on SMM. Patient portals could be used to address risky sexual behaviors; however, past studies have not looked at service design and consumer adoption models for patient-facing IT solutions. Therefore, we created a brief instrument to enable the measurement of attitudes toward using patient portals for STI prevention and care among SMM with access to a patient portal. The instrument was adapted from earlier scientific work, is short, and may be added to health questionnaires focused on sexual health–related technology use. Furthermore, we demonstrated that AMIS-PPSHI might be adapted to include novel consumer-oriented features as technology evolves.

The resulting instrument consists of 12 items and 3 subscales measuring constructs of (1) sexual health engagement and awareness, (2) enhancing dyadic communication, and (3) managing sexual health care. Constructs cover unique aspects of patient portal use. First, portals should communicate personalized sexual health information to the user. Second, interpersonal use of patient portals may occur outside of health settings, particularly in events that share test histories with sexual partners. Third, patient portals should empower patients to engage with an array of sexual health care management services, such as testing and telehealth.

### A Closer Look at PPSHI

PPSHI and its 3 subscales had a strong overall internal consistency. As expected, younger participants are more receptive to technology use. Scores have an indirect relationship with age; SMM aged 15-24 years had the highest scores. Scores stratified by STI diagnosis reveal interesting dynamics, most notably in the enhancing dyadic communication subscale. Scores on the enhancing dyadic communication subscale are the lowest among participants with HIV, an indicator of the highly stigmatized nature of HIV. Interpersonal use of patient portals with sexual partners may likely be lower for participants with HIV or other chronic STIs. Interventions are needed to reduce this stigma and to strengthen self-efficacy for discussing with partners the topics pertinent to sexual health and wellness.

Similar scores by anticipated health care stigma may be an indicator of acceptability of patient portals among participants who may be less likely to receive HIV care services [39,41]. Slightly higher AMIS-PPSHI scores among participants with mental health illness support the acceptability of the patient portal use among patients with mental health disorders [66]. Together with earlier studies, findings support patient portals as a promising avenue to plan interventions around increasing health engagement among marginalized groups, including persons with HIV [28,30,46]. The overall high mean sample score on AMIS-PPSHI may also indicate that patient portal interventions may extend to other areas of health care engagement, such as achieving hepatitis A and B vaccination and screening recommendations for SMM [67-69]. Messages delivered through patient portals have been demonstrated to increase herpes zoster vaccination in adults [70]. Thus, the current global climate of hepatitis A outbreak among SMM reflects missed opportunities to leverage patient portals to deliver hepatitis A and B vaccination screening messages to SMM [71-75]. The application of machine learning algorithms to identify PrEP candidates using EHR data may also be applied to identify candidates for hepatitis A and B vaccination; however, research is needed to develop efficacious algorithms [76].

### Strengths and Limitations

The strength of AMIS-PPSHI is that it is based on empirical research, and it is very timely to the growing technology-based STI prevention models. The scale is shortened in item numbers and response options and still holds a strong internal consistency. The factor analysis is based on both an acceptable sample size of participants and the number of observed variables. Overall, the instrument performed statistically well in psychometric analysis. The age distribution of the sample offers some comparisons and extrapolation across groups of adolescent, young, and adult SMM. However, neither AMIS-PPSHI nor its subscales have been validated as constructs related to patterns of patient portal use for sexual health and wellness. Future validation studies may explore the relationship of constructs with patterns of patient portal use specific to sexual health and wellness. Clinical researchers may test the enhancing dyadic communication subscale as a determinant of an individual’s likelihood to use PHRs for sharing STI test histories with main and nonmain partners [16]. The subscale may then be applied in clinical settings with decision analytics to identify patients for interventions that are less likely to disclose STI PHRs with partners. More data are needed on patterns of patient portal use for sexual health services such as viewing electronic STI test results, viewing health information on STIs, and ordering medications to prevent and treat STIs.

African American or Black SMM are notably underrepresented in the study sample. Future studies are needed to apply PPSHI across a broad nationwide sample of Black SMM and youth—the race or ethnic group most overburdened by HIV and other STI incidence and prevalence [34,38]. Additional validation studies are needed among other priority populations for STI prevention in the United States and other countries with burgeoning mHealth environments. A further limitation is that the instrument does not include items focused on the use of patient portals to report sexual health–related behaviors and outcomes. Ecological momentary assessment and patient-reported outcome measures are mechanisms that can feed data into patient portal systems and inform decision support algorithms for the user or health care providers [77-79]. Given the adaptability of PPSHI, items may be added to patient-reported outcomes to assess perceptions about reporting personal data to the patient portal for sexual health.

### Conclusions

In summary, we suggest that PPSHI and its components could predict behavioral intentions and patterns for patient portal use for health behaviors related to STI prevention and care among SMM. PPSHI is feasibly adaptable to questionnaires and may have useful applications in electronic patient intake surveys. Short surveys on patient intake forms assessing risk behaviors have been used to inform clinical decision support algorithms, prompting providers to encourage STI screening for patients [80]. Assessing PPSHI constructs in patients may similarly be useful in informing provider messaging within decision support algorithms, for example, encouraging patients to share STI PHRs with partners.

## Appendix

Multimedia Appendix 1 Electronic Sexual Health Information Notification and Education (eSHINE) Study Patient Portal Instrument, 2014-2016, N=35.

## Abbreviations

AMIS: American Men’s Internet Survey
CFA: confirmatory factor analysis
CFI: comparative fit index
EFA: exploratory factor analysis
EHR: electronic health record
eSHINE: Electronic Sexual Health Information Notification and Education
Health IT: Health information technology
HINTS: Health Information National Trends Survey
KMO: Kaiser-Meyer-Olkin
K6: Kessler 6-item psychological distress scale
mHealth: mobile health
ONC: Office of the National Coordinator for Health Information Technology
PCA: principal component analysis
PHR: personal health record
PPI: patient portal instrument
PPSHI: patient portal sexual health instrument
PrEP: pre-exposure prophylaxis
RMSEA: root mean square error of approximation
SMM: sexual minority men
SRMR: standardized root mean square residual
STD: sexually transmitted disease
STI: sexually transmitted infection
TLI: Tucker-Lewis index
