# Correction: Using the Patient Portal Sexual Health Instrument in Surveys and Patient Questionnaires Among Sexual Minority Men in the United States: Cross-sectional Psychometric Validation Study

**DOI:** 10.2196/28358

**Published:** 2021-03-05

**Authors:** Kevon-Mark P Jackman, Jeremy Kane, Hadi Kharrazi, Renee M Johnson, Carl Latkin

**Affiliations:** 1 Department of Mental Health Johns Hopkins Bloomberg School of Public Health Baltimore, MD United States; 2 Department of Epidemiology Columbia University Mailman School of Public Health NY, NY United States; 3 Department of Health Policy and Management Center for Population Health IT Johns Hopkins Bloomberg School of Public Health Batimore, MD United States; 4 Department of Health, Behavior, and Society Johns Hopkins Blooomberg School of Public Health Baltimore, MD United States

In “Using the Patient Portal Sexual Health Instrument in Surveys and Patient Questionnaires Among Sexual Minority Men in the United States: Cross-sectional Psychometric Validation Study” (J Med Internet Res 2021;23(2):e18750) four errors were noted.

In the originally published paper, the “greater than or equal to” symbol (≥) was missing in three places in tables and in one place in the main text due to an XML conversion error. The following corrections have been made:

In Table 1, under “Age (years),” “40” has been corrected to “≥40”.

In Table 5, under “Age category,” “40” has been corrected to “≥40”.

In Table 6, under “Age category,” “40” has been corrected to “≥40”.

In the section “AMIS-PPSHI Scores,” the symbol was missing in the following sentence:

However, scores were marginally higher among participants with a Kessler 6-item psychological distress scale (K6) score13.

This has been corrected to:

However, scores were marginally higher among participants with a Kessler 6-item psychological distress scale (K6) score≥13.

The correction will appear in the online version of the paper on the JMIR Publications website on March 5, 2021, together with the publication of this correction notice. Because this was made after submission to PubMed, PubMed Central, and other full-text repositories, the corrected article has also been resubmitted to those repositories.

