# Supplementary material for: Using the Patient Portal Sexual Health Instrument in Surveys and Patient Questionnaires Among Sexual Minority Men in the United States: Cross-sectional Psychometric Validation Study
Source: J Med Internet Res. 2021 Feb 10;23(2):e18750. doi: 10.2196/18750 (PMC7935249; doi:10.2196/18750)
Supplement: Multimedia Appendix 1 [file jmir_v23i2e18750_app1.docx]

| **Multimedia Appendix 1. Electronic Sexual Health Information Notification and Education (eSHINE) Study Patient Portal Instrument, 2014-2016, N=354** | | | |
| --- | --- | --- | --- |
| **#** | **Item** | **Factor Loadings** | **Subscale Name & Cronbach’s alpha** |
| **1** | PHRs are a more convenient way to manage my health records. (7-point; strongly disagree to strongly agree) | 0.7820 | **Subscale: Sexual Health Engagement**  **Reliability coefficient: 0.82** |
| **2** | PHRs encourage people to be more aware of their health. (7-point; strongly disagree to strongly agree) | 0.7132 |  |
| **3** | PHRs will help people like me make better health decisions. (7-point; strongly disagree to strongly agree) | 0.6529 |  |
| **4** | I plan to manage my medical records with PHRs in the future. (7-point; strongly disagree to strongly agree) | 0.6500 |  |
| **5** | If given a PHR, how likely would you use to look up information on the following topics: Medications to treat STDs. (7-point; very unlikely to very likely) | 0.8167 | **Subscale: Informational Resource Compatibility**  **Reliability coefficient: 0.88** |
| **6** | If given a PHR, how likely would you use to look up information on the following topics: Medication to prevent STDs. (7-point; very unlikely to very likely) | 0.8242 |  |
| **7** | If given a PHR, how likely would you use to look up information on the following topics: How STDs are transmitted. (7-point; very unlikely to very likely) | 0.7193 |  |
| **8** | In addition to electronic STD results, which services are important for PHRs to include: Tips/tools for managing sexual health (7-point; Not at all important to extremely important) | 0.8936 | **Subscale: Valuation of Services**  **Reliability coefficient: 0.91** |
| **9** | In addition to electronic STD results, which services are important for PHRs to include: Locating services STD test centers. (7-point; Not at all important to extremely important) | 0.8535 |  |
| **10** | In addition to electronic STD results, which services are important for PHRs to include: Services to communicate with your doctor or health professionals. (7-point; Not at all important to extremely important) | 0.7977 |  |
| **11** | In addition to electronic STD results, which services are important for PHRs to include: Counsel and resources for people with STDs. (7-point; Not at all important to extremely important) | 0.7906 |  |
| **12** | In addition to electronic STD results, which services are important for PHRs to include: Access to all of your medical records. (7-point; Not at all important to extremely important) | 0.6712 |  |
| **13** | How will PHRs affect: Control over my sexual health and decision making. (7-pooint; very harmful to very helpful) | 0.7675 | **Subscale: PHR Impact**  **Reliability coefficient: 0.85** |
| **14** | How will PHRs affect: Confidence in the testing information a partner shares with me. (7-pooint; very harmful to very helpful) | 0.8031 |  |
| **15** | How will PHRs affect: Communication between my partner(s) and myself. (7-pooint; very harmful to very helpful) | 0.8008 |  |
| **16** | PHRs make it easier for people to routinely have "check in" conversations with partners about STI prevention. (7-point; strongly disagree to strongly agree) | 0.6835 |  |
| **17** | Partners using PHRs will start talking about STI prevention EARLIER in a relationship. (7-point; strongly disagree to strongly agree) | 0.6335 |  |
| **18** | I would have more discussions with partners about STI testing if PHRs were more commonly used. (7-point; strongly disagree to strongly agree) | 0.5405 |  |
| **19** | Using PHRs with a partner builds trust (7-point; strongly disagree to strongly agree) | 0.6454 |  |
| Sources:  1) Jackman KP, Hightow-Weidman L, Poteat T, Wirtz AL, Kane JC, Baral SD. Evaluating psychometric determinants of willingness to adopt sexual health patient portal services among black college students: A mixed-methods approach. J Am Coll Health. 2019 Nov 11:1-8. doi: 10.1080/07448481.2019.1660352. Epub ahead of print. PMID: 31710578; PMCID: PMC7211543.  2) Jackman KP, Murray S, Hightow-Weidman L, Trent ME, Wirtz AL, Baral SD, Jennings JM. Digital technology to address HIV and other sexually transmitted infection disparities: Intentions to disclose online personal health records to sex partners among students at a historically Black college. PLoS One. 2020 Aug 21;15(8):e0237648. doi: 10.1371/journal.pone.0237648. PMID: 32822360; PMCID: PMC7442257. | | | |
